# Translational medicine: science or wishful thinking?

**DOI:** 10.1186/1479-5876-6-31

**Published:** 2008-06-17

**Authors:** Martin Wehling

**Affiliations:** 1 Clinical Pharmacology Mannheim, University of Heidelberg, Mannheim, Germany

## Abstract

"Translational medicine" as a fashionable term is being increasingly used to describe the wish of biomedical researchers to ultimately help patients. Despite increased efforts and investments into R&D, the output of novel medicines has been declining dramatically over the past years. Improvement of translation is thought to become a remedy as one of the reasons for this widening gap between input and output is the difficult transition between preclinical ("basic") and clinical stages in the R&D process. Animal experiments, test tube analyses and early human trials do simply not reflect the patient situation well enough to reliably predict efficacy and safety of a novel compound or device. This goal, however, can only be achieved if the translational processes are scientifically backed up by robust methods some of which still need to be developed. This mainly relates to biomarker development and predictivity assessment, biostatistical methods, smart and accelerated early human study designs and decision algorithms among other features. It is therefore claimed that a new science needs to be developed called 'translational science in medicine'.

## Introduction

These days, "translational medicine" is a fashionable term describing the inclination of biomedical researchers to ultimately help patients.

As if this wish would be novel, it is being increasingly used by scientific funding agencies (e.g. NIH, European Union framework programs), regulatory authorities (e.g. FDA), researchers and patient care providers.

This inflationary use has been triggered by a simple and powerful fact: despite increased efforts and investments into R&D, the output of novel medicines has been declining dramatically over the past years [1]. One of the reasons for this widening gap between input and output is the difficult transition between preclinical ("basic") and clinical stages in the R&D process. Animal experiments, test tube analyses and early human trials do simply not reflect the patient situation well enough to reliably predict efficacy and safety of a novel compound or device.

Public and private responses in reflection of this observation have been numerous during the past years. This includes the "Critical Path" initiative by the FDA [2], the re-orientation of the NIH ("road-map"), enforced by Dr Zerhouni's leadership towards translational medicine with an estimated 10 billion USD channeled into translational medicine centers and other activities; major drug companies and US universities (e.g. Duke or UPenn) are being driven to establish translational medicine departments or at least working groups.

## Measures and obstacles for translational medicine

So far, so good. But what has really happened and how do we expect it to affect future R&D outcome?

Both in academia and industry, the wish to translate better has increased the awareness for interface problems; in academia, more clinical trials shall be performed as the tougher variant of medical research if compared with test tube research. Clinical trials require a lot more resources, paper work and endurance, and the rewards are still smaller in the public appreciation (papers, impact factors). This challenge has been identified and a huge amount of money is now supplied to investigator driven trials in academia, especially through the translational medicine centers by the NIH. The topic as such has been reflected in numerous reviews and opinion papers, e.g. by Sonntag [3] or Littman et al. [4] who in particular reflect on the various hurdles opposing the progress of translational medicine such as scientific, financial, ethical, regulatory, legislative and practical problems. It should be noted though that in Europe, investigator initiated trials (IITs) as the most important manifestation of clinical translational efforts in academia, are still rare and increasingly burdened by legislatory (European drug laws, especially the 12^th ^revision of the German 'Arzneimittelgesetz') and financial constraints.

In industry, as both preclinical and clinical studies have been performed routinely and professionally long before this call for translational activities emerged, now the major task is the intertwining and alignment of preclinical and clinical studies along the artificially straight drug discovery and development process. Here, it is more of an interface problem, than reflecting the lack of access to clinical trials. The main drawback of translational studies and related personalized medicine approaches in industry is the mandatory economical interest which is not in line with all sophistications of drug profiling leading to narrowed windows of opportunities. It seems that in some instances, such translational efforts would have to come from parallel, independent, presumably academic IITs.

In public science funding calls (e.g. EU Framework Program 7) or scientific meetings (e.g. Endocrine Society Meeting), "translational" seems to identify any proposal or topic which involves clinical material and non-established mechanistic approaches or early compound/medical device testing. Even epidemiology is coined as a translational medicine tool, if patient data are analyzed.

Do those activities and interpretations of translational medicine live up to the expectations raised by the original motivation, namely to ultimately help patients?

The threat to increased output is the fact that most activities under the umbrella "translational medicine" are pretentious and reflect phraseology, thus just wishful thinking.

Translational efforts are as old as medicine; all drugs on the market had a successful translational process in their history, and the wish to help patients by scientific tools has been around for as long as medical science exists. If the pressure exerted by lacking success just induces new terminology for old processes, it is simply not enough to warrant a major change. If success will not show, however, biomedical science could even become a major loser in the battle on investments into the future of mankind, given e.g. the environmental and energy supply threats.

## How to develop translational science in medicine?

What is lacking to ensure success, sustainability and substantial contributions of translational medicine?

As with all science, methods, systematic approaches and tools are clues for success. This certainly applies to translational medicine. At present, the major weakness in its fashionable surge is the *lack of a scientific backbone*. In academia, translational programs to take new compounds or devices systematically and under translational auspices into man are almost completely lacking, and pathophysiological studies of questionable relevance are rather being induced by the new supply of money and increased pressure to yield useful results. This is no true translation from preclinical to clinical stages, thus, the translational aspect is virtually lacking. In industry, better translation as a goal is not supported by better tools or methods to improve the translational process which had already been in place (driven by necessity).

Those *methods and tools *to facilitate the translational process *need urgently to be developed*. A major aspect in this regard is the description and assessment of key indicators in a translational process, so-called biomarkers, which are needed for translational prediction. They are the main elements in predicting efficacy and safety from animal to man and could be seen to be accountable for 80–90% of translational success. But they are very different. Some do not even exist in humans (e.g. certain hormones), or cannot be measured ethically (e.g. serial brain slices). This implies that their values to predict translational outcomes are also very different, and a scoring system for the predictive value seems to represent a necessary tool in this process. The first proposed quantitative biomarker scoring system depends on 10 simple questions about e.g. accuracy, reproducibility, accessibility and human experience for a given biomarker [5]. If applied to imaging biomarkers, this early system seemed to produce reasonable scores if compared to "gut feeling" scores which are the only ones available so far [6]. Those biomarkers, notabene, are the major tools in pharmaceutical R&D to govern decisions worth 100–300 million USD (e.g. investing in major phase III clinical trials). It is obvious that the importance of biomarkers or other tools of translational science do not only apply to pharmaceutical development, but are essential to translational processes of medical devices or even procedural (e.g. surgical) developments.

This is just one example for an emerging tool in the nascent science of translation. Many areas need development alike including biostatistics (e.g. how to deal with multiple read-out situations from expression analyses, gene SNP analyses or serum markers panels), smart early clinical trial design (this has been facilitated by microdosing techniques and experimental IND [Investigational New Drug] applications) and the discovery of novel biomarkers (not just grading of existing ones) fit for translational purposes. Descriptive studies are relevant to translational medicine in that they may point to essential hypothesis and novel targets [7]. Human genetics provides powerful tools for target validation to reflect back on preclinical research, especially animal model development and its routine application to target validation needs standardization and refinement.

Imaging seems most promising in this regard as it is a noninvasive and very informative tool which can be applied across species including man, but this certainly is not the answer to all needs. In terms of lacking biomarkers, safety biomarkers seem to be most antiquated (e.g. liver transaminases which have been used for 50 years in safety assessments), and certainly need fresh "injections" most urgently.

The art of designing a translational medicine plan in drug development can be hardly learned by a scientist who has not gathered wide, concomitant preclinical and clinical experiences. However, those scientists are still as rare as natural diamonds; thus, education, breeding the translational scientist to be fit for purpose, would be a central feature of this science as of any science.

Essential requirements for such plans should be defined and a reproducible and widely applicable structure developed. The stop/go-decision reasoning in the development process of drugs and non-drug innovations would gain standardization, reproducibility and, thereby, increased reliability. In this act, the assessment of biomarker validity would represent an essential feature, and the construct of smart clinical early trials would be build around those capacities of developed and selected biomarker settings.

This list of tools (table 1) is by far not complete; it would just name major elements which urgently need development and could finally lead to something which would deserve the term "translational science in medicine".

**Table 1:** Tools of Translational Science in Medicine as backbone of an emerging science

New biomarker development, e.g. imaging or serum parameters
Translational toxicology including more powerful biomarkers
Biomarker scoring systems to grade their predictive potency
Smart, early human study design, including novel approaches e.g. microdosing and descriptive trials
Biostatistics development to cope with multiple read-out problems and small human studies
Human genetics

This science could be the most important novel medical discipline in terms of patients' health to be developed in the future. If successful, it should not only help individual patients by novel diagnostic and treatment options, but also prevent biomedicine from shrinking into oblivion after decades of lack of success.

## Competing interests

The author was employed by AstraZeneca R&D, Mölndal, as director of discovery medicine (= translational medicine) from 2003–2006, while on sabbatical leave from his professorship at the University of Heidelberg. After return to this position in January 2007, he receives lecturing and consulting fees from Pfizer, Roche, Daiichi-Sankyo, Novartis, PAION and Lilly.
